# Mass spectrometry imaging as an emerging tool for studying metabolism in human brain organoids

**DOI:** 10.3389/fmolb.2023.1181965

**Published:** 2023-05-22

**Authors:** Gerarda Cappuccio, Saleh M. Khalil, Sivan Osenberg, Feng Li, Mirjana Maletic-Savatic

**Affiliations:** ^1^ Department of Pediatrics–Neurology, Baylor College of Medicine, Houston, TX, United States; ^2^ Jan and Dan Duncan Neurological Research Institute, Texas Children’s Hospital, Houston, TX, United States; ^3^ Department of Pathology and Immunology, Baylor College of Medicine, Houston, TX, United States; ^4^ Center for Drug Discovery, Baylor College of Medicine, Houston, TX, United States; ^5^ Department of Neuroscience, Baylor College of Medicine, Houston, TX, United States

**Keywords:** metabolome, neurons, neuroprogenitors, brain organoids, mass spectrometry imaging method

## Abstract

Human brain organoids are emerging models to study human brain development and pathology as they recapitulate the development and characteristics of major neural cell types, and enable manipulation through an *in vitro* system. Over the past decade, with the advent of spatial technologies, mass spectrometry imaging (MSI) has become a prominent tool for metabolic microscopy, providing label-free, non-targeted molecular and spatial distribution information of the metabolites within tissue, including lipids. This technology has never been used for studies of brain organoids and here, we set out to develop a standardized protocol for preparation and mass spectrometry imaging of human brain organoids. We present an optimized and validated sample preparation protocol, including sample fixation, optimal embedding solution, homogenous deposition of matrices, data acquisition and processing to maximize the molecular information derived from mass spectrometry imaging. We focus on lipids in organoids, as they play critical roles during cellular and brain development. Using high spatial and mass resolution in positive- and negative-ion modes, we detected 260 lipids in the organoids. Seven of them were uniquely localized within the neurogenic niches or rosettes as confirmed by histology, suggesting their importance for neuroprogenitor proliferation. We observed a particularly striking distribution of ceramide-phosphoethanolamine CerPE 36:1; O2 which was restricted within rosettes and of phosphatidyl-ethanolamine PE 38:3, which was distributed throughout the organoid tissue but not in rosettes. This suggests that ceramide in this particular lipid species might be important for neuroprogenitor biology, while its removal may be important for terminal differentiation of their progeny. Overall, our study establishes the first optimized experimental pipeline and data processing strategy for mass spectrometry imaging of human brain organoids, allowing direct comparison of lipid signal intensities and distributions in these tissues. Further, our data shed new light on the complex processes that govern brain development by identifying specific lipid signatures that may play a role in cell fate trajectories. Mass spectrometry imaging thus has great potential in advancing our understanding of early brain development as well as disease modeling and drug discovery.

## Introduction

Organoid culture systems are organ-specific, self-assembling, three-dimensional models derived from primary tissue stem cells, embryonic stem cells, or induced pluripotent stem cells (iPSCs) (Sasai, 2013; Clevers, 2016; Takebe and Wells, 2019; Yoon et al., 2019; Artegiani et al., 2020). Multiple organoid differentiation methods have been developed to generate organoids for different tissues including cerebral organoids (Lancaster and Knoblich, 2014; Paşca et al., 2015), based on the timely administration of small molecules and growth factors to promote the formation of the desired brain regions such as the forebrain, midbrain (Jo et al., 2016; Monzel et al., 2017), cerebellum (Muguruma et al., 2015), hippocampus, retina (Eiraku et al., 2011), and others. Fundamental organization of the developing brain is orchestrated by thousands of molecules simultaneously. To better understand the mechanisms of brain development in both physiological and pathological conditions (Uzquiano et al., 2022), this complex cellular and molecular milieu can be studied using multi-omics platforms such as proteomics, transcriptomics, and metabolomics (Shou et al., 2020; Qian et al., 2019).

One of the new technologies not yet utilized for brain organoid studies is mass spectrometry imaging (MSI)—or imaging mass spectrometry—that uses mass spectrometry to directly resolve information on the molecular composition of complex biological samples in a single experiment (Watrous et al., 2011; Seeley et al., 2011; Caprioli RM, 2016; Tang et al., 2018; Chen et al., 2019; Spruill et al., 2022). MSI has emerged as a powerful molecular imaging technology, bringing unparalleled molecular specificity and sensitivity to map the distribution of small molecules in tissues. While conventional mass spectrometry has revolutionized the world of ‘omics sciences, drug development and metabolism, it has been mostly used for *in vitro* studies of analytes that have been extracted from fluids or tissues (Arnold et al., 2015). MSI, on the other hand, uses existing mass spectrometry detection platforms to map simultaneously spatially-resolved distributions of hundreds to thousands of label-free small biomolecules from a single tissue section *in situ*, without any need for specific molecular probes, bringing new dimensions to molecular imaging and placing it at the forefront for many applications in metabolism and small molecule/drug discovery research (Shariatgorji et al., 2014; Groseclose et al., 2015; Groseclose and Castellino, 2013; Goodwin et al., 2017). Thus, unlike traditional liquid chromatography-mass spectrometry (LC-MS), MSI can determine the spatial distribution and topographic organization of many different molecular analytes in tissue sections such as lipids, peptides, amino acids, as well as drugs and drug metabolites, without labeling or staining and despite the sample heterogeneity. The spatial resolution of metabolites approaches the dimensions of mammalian cells, so tissues with multiple cell types or genetic chimera can give very different metabolic signals from adjacent pixels.

Furthermore, to ascertain the localization of specific compounds in tissue samples, MSI images can be overlaid onto histological images obtained from the same or adjacent tissue sections (Miura et al., 2010). This technology adds to the histologist’s toolbox: it complements it without replacing it. By integrating microscopy with MSI, the applications are almost limitless and could be used in a variety of biologically and medically relevant research studies. Given these features, MSI can contribute to studies of the molecular basis of diseases, provide insights into mechanisms, and integrate tissue morphology with information at the molecular level. Thus, it can facilitate a deeper understanding of the connections between the genome, phenotypic features, and biological responses to the environment and other stimuli (Choi et al., 2018; Kandel et al., 2022).

In addition, the pharmaceutical industry has taken advantage of MSI to enable an array of high throughput screening modalities for pharmaceutical assessments. There, obtaining information about the absorption, distribution, metabolism, and elimination of a new lead compound via a pharmacokinetic study in a preclinical model is often the first step towards understanding the *in vivo* properties of a drug-like molecule in humans. MSI can provide not only reliable, label-free qualitative and quantitative distribution information for a compound of interest but also, at the same time, detect its bio-transformed metabolites (Spruill et al., 2022). This is relevant for both determining which compounds are truly active and which may have potential toxic effects.

So far, MALDI (matrix-assisted laser desorption/ionization), DESI (desorption electrospray ionization) and SIMS (secondary ion mass spectrometry) are the most popular methods for MSI (Chughtai and Heeren, 2010). Among those, SIMS can provide superior spatial resolution, but the high energy of the ion beam leads to severe fragmentation of analytes. DESI is a soft ionization method operating under ambient conditions. Its spatial resolution, however, is limited to about 35 µm (Campbell et al., 2012). Until today, MALDI is the most widely used soft ionization technique for imaging. It can handle a wide mass range, offers direct analysis of analytes, and is versatile with respect to different tissue-specific chemical compounds (O’Connor and Costello, 2001).

Given all the advantages of MALDI-MSI and the lack of its utilization for studies of human brain organoid models, we here provide a comprehensive protocol for sample preparation for the analysis of brain organoids via high resolution-MALDI-MSI (HR-MALDI-MSI). We demonstrate the power of HR-MALDI-MS by focusing on lipids and showing differences in lipid distribution in specific and functionally different areas of brain organoids, potentially critical for cell function and organoid maturation.

## Methods

### Human dorsal forebrain organoids

To generate dorsal human forebrain organoids, we adapted the Pasca protocol for its advantages (Pașca et al., 2019): 1) It recapitulates with considerable accuracy the development of the human cortex and the organization of neuroprogenitor zones (Paşca et al., 2015; Qian et al., 2019; Birey et al., 2017); 2) It has been widely used, leading to numerous publications that have characterized it thoroughly (Birey et al., 2017; Liu et al., 2020; Cao et al., 2017; Madelaine et al., 2017; Trevino et al., 2020; Pașca et al., 2019; Uzquiano et al., 2022) 3) It is highly efficient and consistent, resulting in highly reproducible organoids both within and between experiments (Yoon et al., 2019), and 4) It can be easily scaled-up to generate large numbers (thousands) of organoids, allowing us to produce sufficient material in a short period of time to perform comprehensive analyses. In this organoid model, iPSC cultures are subsequently transferred into AggreWell™800 Microwell Culture Plates to develop about 300 embryoid bodies. To achieve rapid and efficient neural induction, both the Bone morphogenetic proteins (BMP) and transforming growth factor beta (TGF-β) signaling pathways are inhibited with small molecules, dorsomorphin and SB-431542, respectively.

Here, we used induced pluripotent stem cells (iPSCs) generated by reprogramming of control fibroblast cells (GM01888, https://catalog.coriell.org/0/Sections/Search/Sample_Detail.aspx?Ref=GM01888&Product=CC) with Sendai virus (Chen et al., 2013). We assessed the derived iPSC pluripotency using Karyostat, pluripotency score (Thermo Fisher), alkaline phosphatase staining (Vector^®^ Red Substrate Kit, Alkaline Phosphatase, SK-5100), and differentiation into each of the three germ layers (Human Pluripotent Stem Cell Functional Identification Kit, R&D system Catalog #: SC027B).

iPSCs of high purity were placed in the AggreWell plate and on day eight, the floating spheroids were moved to suspension dishes and serum-free media containing fibroblast growth factor 2 (FGF2) and ascorbic acid. To promote differentiation of neuroprogenitors into neurons, FGF2 was replaced with brain-derived neurotrophic factor (BDNF) and neurotrophic factor 3 (NT3) starting on day 22–60, after which we used only neural medium without growth factors. Using this method, we can produce functional organoids with mature neurons and glial cells, preserving tissue architecture and orientation (Manganas et al., 2021).

### Brain organoid section preparation

**FIGURE 1:**
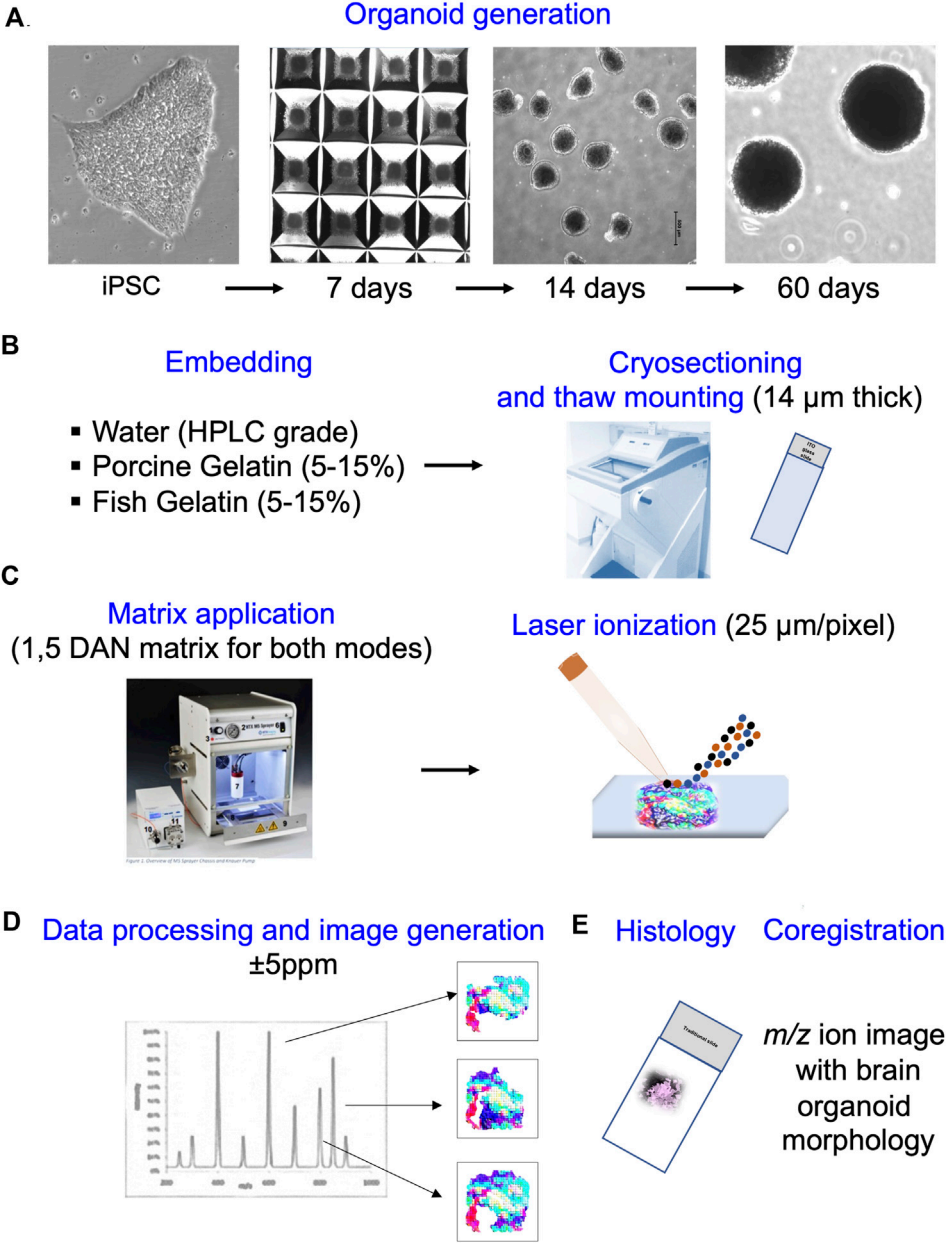
Overall workflow of high-resolution matrix-assisted laser desorption/ionization mass-spectrometry imaging (HR-MALDI-MSI). **(A)** Human dorsal forebrain organoids were generated from induced pluripotent stem cells (iPSCs) over the course of 60 days. **(B)** Brain organoids were embedded in water, porcine gelatin, or fish gelatin, cryo-sectioned into 14 µm sections, and thaw mounted on indium tin oxide coated (ITO slides) for MSI. Slides were stored at −80°C. **(C)** To prepare for MSI, 1,5 DAN matrix was applied on top of sections to help ionize the lipids in both positive and negative ion modes, with stage step size of 25 µm per pixel. One full-scan Fourier Transform MS (FTMS) acquired from each 25 µm × 25 µm sampling region corresponds to pixel size of the MSI data. **(D)** From each laser position (FTMS), a mass spectrum is generated and analyte distribution images created depending on the analyte signal intensities from FTMS position-specific data. **(E)** Adjacent sections mounted onto a traditional glass slide were used for histological staining to co-register the ion image with brain organoid anatomy.

Brain organoids derived from iPSC were collected at 60 days using wide bore tips (ART™ Wide Bore Filtered Pipette Tips) (Figure 1). They were washed three times with 1× Dulbecco’s phosphate-buffered saline (DPBS), without CaCl_2_ and MgCl_2_ (Life Technologies, cat. no. 14190-144) and then with distilled water. The organoids were then put firmly in the center of the Cryomolds^®^ cast blocks (25 mm × 20 mm x 5 mm) and embedding solution (10% of gelatin from fish (Sigma-Aldrich G7041-100G) or porcine skin (Sigma-Aldrich, cat. no. G1890), or culture-grade distilled water) was poured over and allowed to solidify (Figure 1). 10% of gelatin solutions were prepared by stirring and heating the respective gelatin at 70°C–80°C for 2 h, and then moving to 37°C incubator for 30 min to remove bubbles and to equilibrate to the temperature of the incubator where organoids were grown. The molds with the organoid–gelatin/water suspension were rapidly frozen by placing them molds into a Petri dish sitting on dry ice and containing cold 100% ethanol. When completely frozen molds, evident by a change in color to solid white, the organoid–gelatin/water blocks were taken out and stored in aluminum foil or tin cups at −80°C. Tissue samples were cut in 14 μm sections with a cryotome at −20°C to −25°C. Structurally uniform sections were thaw-mounted onto glass slides and indium tin oxide (ITO) conductive–coated slides (Hudson Surface Technology, New York, United States) (Figure 1).

### Slide matrix deposition for MALDI-MSI

Tissue sections were put from the −80°C directly into a desiccator for 20 min to minimize condensation of atmospheric water on their surfaces. For MSI in positive and negative ion modes, 1,5 Diaminonaphthalene (DAN) matrix (9 mg/mL in 50% acetonitrile (ACN) and 0.4% N,N-Dimethylformamide (DMF)) was sprayed with the HTX M5+ sprayer (HTX Technologies LLC, Carrboro), respectively (Figure 1). Spraying parameters were as follows: temperature = 65°C, nozzle velocity = 1,200 mm/min, pump flow rate = 100 μL/min, number of passes = 4, track spacing = 2.5 mm, and 10 psi nitrogen gas pressure. The amount of matrix (Wm) deposited was 0.00055 mg/mm^2^ of tissue area and the linear flow rate was 0.00008 mL/min.

### MSI instrumentation

A high resolution MSI platform for visualizing brain organoid lipids was implemented by mounting a MALDI ion source containing a dual-ion funnel interface (Spectroglyph LLC, United States) to a Q-Exactive mass spectrometer (Thermo Fisher Scientific, Massachusetts, United States) (Belov et al., 2017; Ellis et al., 2018). An attached Q-switched frequency-tripled Nd:YLF laser of 349-nm wavelength was used at a repetition rate of 1 KHz and pulse energy of ∼1.3–1.4 µJ. The laser was focused and controlled to a spot size of ∼15 µm in diameter. The sample was attached to the MALDI injector stage and the high-pressure ion funnel was maintained at 7.4–7.5 Torr. The low-pressure ion funnel was maintained at 1.6–1.8 Torr. We applied 604 kHz, 80 V0-peak and 780 kHz, 191 V0-peak radio frequency voltages to the high and low-pressure ion funnels, respectively. The Orbitrap mass spectrometry was operated at ion injection time of 250 ms and Fourier Transform MS (FTMS) spectra were acquired in a profile mode using a target mass resolution of 70,000 (Full Width Half Maximum (FWHM) at m/z 400, m/z = mass-to-charge ratio). During MALDI imaging, mass spectrometer starts to acquire data after switching on to contact closure. The signal is sent from the MALDI injector and communicated via the ‘Peripheral Control’ input connection at the side of the Q Exactive MS, and the automatic gain control was switched off.

### MSI acquisition and data analysis

Data were acquired in the m/z range of 200–1,000 and brain organoid image generation was done using an HR-MALDI-MSI system in both positive and negative ion modes (Figure 1). Samples were scanned with lateral resolution of 25 µm pixel size for 14 µm organoid sections of 2–4 mm (h) x 1–1.5 mm (w) size. The Q Exactive MS was operated in positive-ion mode with the mass range of m/z = 400-1,000 whereas in negative ion mode, the mass range was m/z = 200-1,000. DAN matrix peaks were used for internal calibration resulting in a mass accuracy of better than ±5 ppm. The laser beam was focused carefully to avoid oversampling. Automatic gain control was turned off and C-trap injection time was fixed to 250 ms.

SCiLS Lab software version 2022b (SCiLS GmbH, Bremen, Germany) was used for processing of MSI data. Thermo Q Exactive MS spectral data were first imported into SCiLS Lab followed by baseline correction (convolution algorithm) and total ion count (TIC) normalization (Claes et al., 2023). This software package was used to generate mass images from raw data files with a bin width of ∆m/z = 0.01 or ±5 ppm to discriminate m/z images based on mass defect and pixel coverage. Mass spectra from 25 µm/pixel were obtained and all lipid ions were assigned within a mass accuracy of ±5 ppm. False color images (Jet) or RGB (red-green-blue) images were generated from individual lipid ion species. Theoretical m/z values of each lipid species were obtained from the database (www.lipidmaps.org) and ALEX123 lipid calculator (http://alex123.info/ALEX123/MS.php), which were used for image generation with a bin width of ±5 ppm relative to the theoretical value. Various lipid species in the brain organoids were detected and identified based on the exact masses of lipid molecules.

### Brain organoid anatomy

Classical hematoxylin/eosin (HE) staining and immunostainings of adjacent sections were used to correlate the MSI images with the organoid anatomy (Figure 1). The tissues from the adjacent MSI-sections were visualized at ×10 and ×63 magnification using Leica (SP8X) fluorescence microscope and Nikon spinning disc microscope.

*Hematoxylin eosin staining (HE).* Histological sections extracted from each block underwent routine HE staining following standard protocol (Wittekind, 2003). We used commercially available Instant Hematoxylin from Thermo Scientific^®^.

*Immunostaining.* Tissue sections were fixed in 150 μL 4% paraformaldehyde per section for 10 min, before incubation with the primary antibodies diluted in 0.3% Triton-X at 4°C overnight (goat polyclonal IgG *DCX* sc-271390 (Santa Cruz), rabbit polyclonal IgG *PAX6* 901301 (Biolegend)). Secondary antibodies were applied at 1:500 dilution for 120 min at room temperature in the dark (Donkey anti-Rabbit IgG (H + L) Highly Cross-Adsorbed Secondary Antibody, Alexa Fluor™ Plus 647, AB_2762835, Donkey Anti-Goat IgG H&L Alexa Fluor^®^ 555 preabsorbed (ab150134)). To stain the nuclei 1 mg/mL 4′,6-diamidino-2-phenylindole (DAPI, Life Tech, 62248) stock solution was diluted in PBS (1:2,000) before adding to the slide. After 10 min, the DAPI was washed off twice with PBS before mounting the coverslips on glass slides with the anti-fading reagent (Molecular probes, P36930**).**

### Chemicals

Water (HPLC grade), acetonitrile (ACN), and N,N-Dimethylformamide (DMF) were obtained from Fisher Scientific (Waltham, MA). Porcine and fish gelatin, and 1,5-Diaminonaphthalene (DAN) ≥99.0% were obtained from Sigma-Aldrich (St. Louis, MIA). Hematoxylin, Eosin, and Entellan were obtained from Thermo Fisher Scientific. All chemicals used in this study were of the highest purity available.

## Results

### MSI protocol suitable for human brain organoids

We have developed a reliable and reproducible sample preparation method for brain organoid metabolic imaging using HR-MALDI-MSI (Figure 1). To reduce analytical artifacts to a minimum, preservation of sample integrity is integrity is critical for MSI. One of the major issues is the choice of fixatives, because different fixatives such as glutaraldehyde, paraformaldehyde, and cryopreservant materials such as sucrose should be avoided as they frequently affect lipids and other metabolites in the tissue. The optimal fixation method for metabolic imaging is flesh freezing in liquid nitrogen, but care needs to be taken to ensure complete freezing while avoiding tissue cracking (Tang et al., 2018). In case of brain organoids that are very small (∼1–2 mm in diameter), submerging them in liquid nitrogen disintegrates them. Thus, a better method to achieve complete freezing with minimal damage to the tissue is to place them directly from the incubator into a warmed embedding solution and to then freeze this suspension in dry ice-cooled ethanol.

**FIGURE 2:**
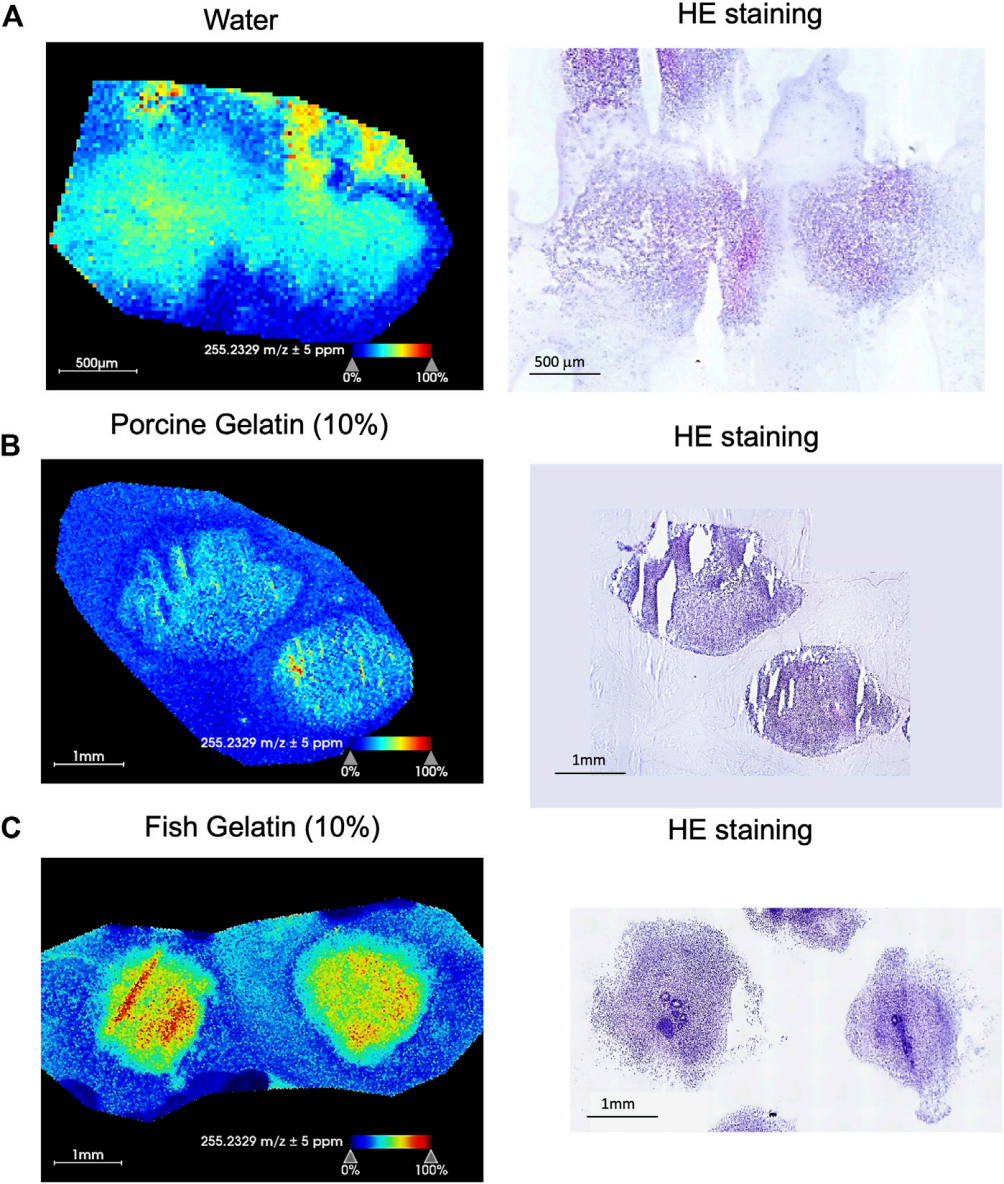
Comparison of brain organoid MSI after embedding in water, porcine gelatin, or fish gelatin. 60-day brain organoids were embedded in water **(A)**, 10% porcine gelatin **(B)**, or 10% fish gelatin **(C)**. Sectioning, mounting, matrix application, and MSI (*left panels*) performed in the same manner for all three embedding media. Adjacent sections were stained with hematoxylin and eosin (HE). Nuclei are stained with hematoxylin (purple) and cytoplasmic components with eosin (pink) (*right panels*). The representative MSI images show distribution of palmitic acid (FA 16:0; m/z 255.2329) in all three cases.

The next step in tissue preparation is embedding the organoids prior to cryo-sectioning. Common embedding materials such as the optimal cutting temperature (OCT) compound and Formalin-Fixed Paraffin Embedding (FFPE) are typically not compatible with MSI since these compounds interfere with matrix deposition and/or ionization (Buchberger et al., 2018). To optimize the embedding material to reduce the fragileness and maintain integrity of the brain organoid slices, we examined three materials: water, 10% gelatin from porcine skin, and 10% gelatin from fish skin (Figure 2). The 60-day-old organoids from the same batch were frozen in cold ethanol on dry ice, embedded in one of the media, and processed for MSI the same way. Specifically, we dry sprayed the sections with DAN matrix to reduce possible delocalization for both positive and negative ion modes. This was performed by reducing the nozzle velocity and pump flow rate that eventually gave a lower linear flow rate with drier spray for increased image resolution. DAN was previously reported as a matrix of choice for MALDI-MSI of phospholipids and small metabolites in both positive and negative ion mode (Korte and Lee, 2014).

Adjacent organoid sections were used for MSI and HE stain to try to correlate analyte distribution with the specific parts of the organoid tissue (Figure 2). To compare the three embedding compounds (water, porcine gelatin, and fish gelatin), we show the distribution of palmitic acid (FA 16:0; m/z 255.2329) in the representative MSI images from organoid sections (left panels) and the respective HE stainings (right panels). Tissue morphology was the worst when water was used as the embedding material (Figure 2A). The organoid tissue contained many holes, suggesting that freezing disrupted the tissue. Embedding the tissue in porcine gelatin produced better results, both in terms of tissue architecture and MSI data, compared to water. However, we observed big gaps in the tissue due to breakage of the organoids and these interfered with the MSI as well (Figure 2B). We obtained the highest yield of MSI data with 10% gelatin from fish skin, including the higher yield of MSI and preservation of tissue morphology as confirmed by HE staining of a serial section (Figure 2C). Therefore, for all ensuing studies, we used only the 10% fish gelatin as an embedding solution.

### MSI detects a variety of lipids in human brain organoids

**FIGURE 3:**
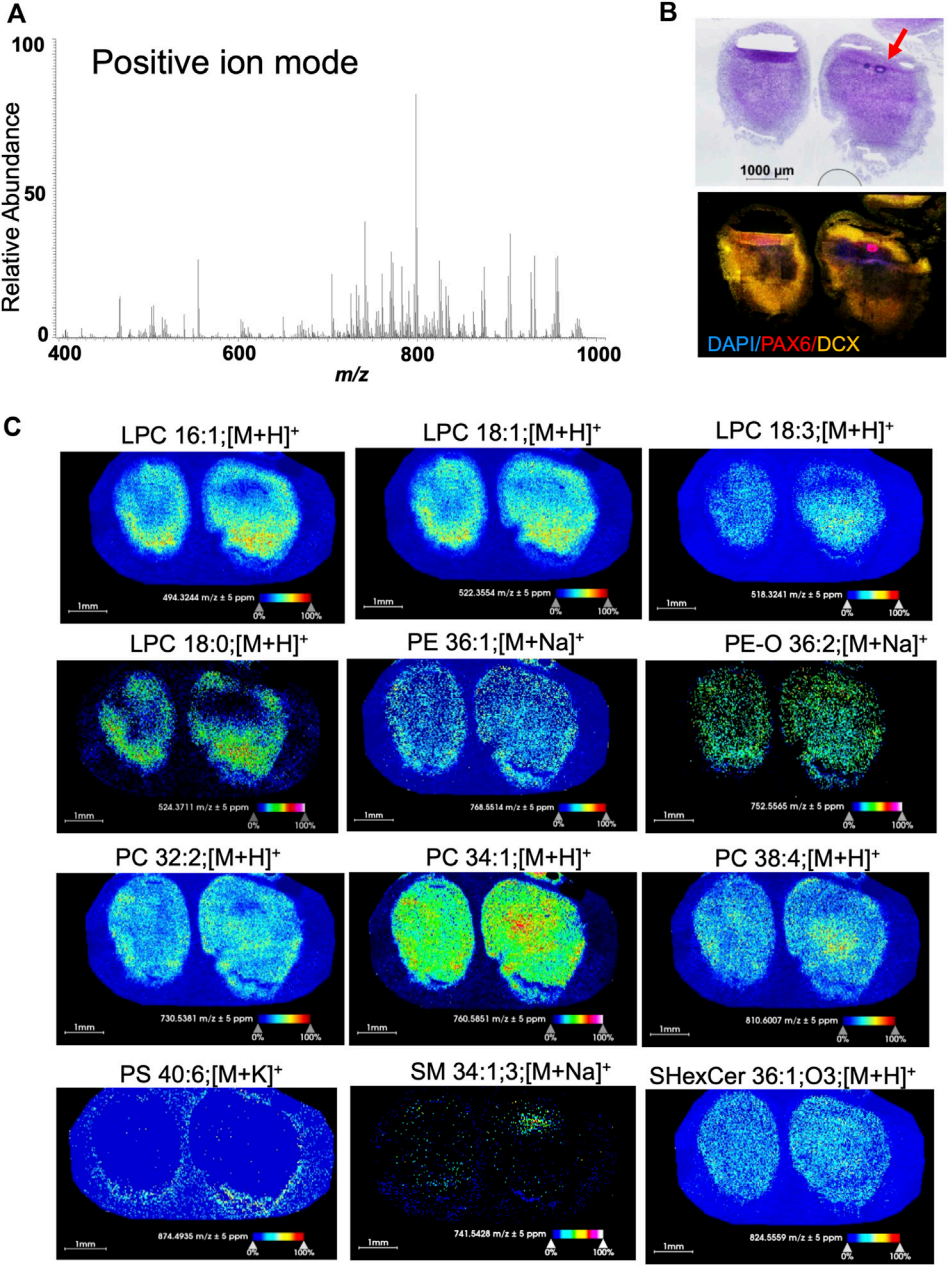
Positive ion mode MSI from 60-day-old brain organoids shows different spatial distribution and abundance of lipid species. **(A)** Average m/z spectra generated from the positive ion mode of 60-day-old brain organoids detected 114 lipid species across the measured mass range of m/z 400-1,000. This included lysolipid (m/z 400–600) and phospholipid (m/z 700–1,000) signals at positive polarity. **(B)** Hematoxylin and eosin (HE) staining along with confocal micrograph of serial sections shows organoid morphology and expression of DAPI (nuclear stain), PAX6 (marker of neuroprogenitors localized in rosettes) and doublecortin (DCX, marker of immature neurons). Arrow points to a rosette. **(C)** Ion distribution images of lipids that are visualized and detected in positive ion mode represent several lipid classes including lysolipids, glycerophospholipids and sphingolipids. All images were generated with a bin width of *Δm/z* = 0.01 at a mass resolving power of 70,000 at m/z = 400 and a mass accuracy within ±5 ppm. The spatial resolution was set to 25 µm/pixel. Each dot on the image is one pixel. The scale bar represents 1 mm and ion intensities are normalized to the total ion count (TIC) and scaled from 0% to 100%. LPC, lysophosphatidyl-choline; PE, phosphatidyl-ethanolamine; PC, phosphocholine; PS, phosphatidyl-serine; SM, sphingomyelin; SHexCer, sulfatide hexosyl ceramide.

**FIGURE 4:**
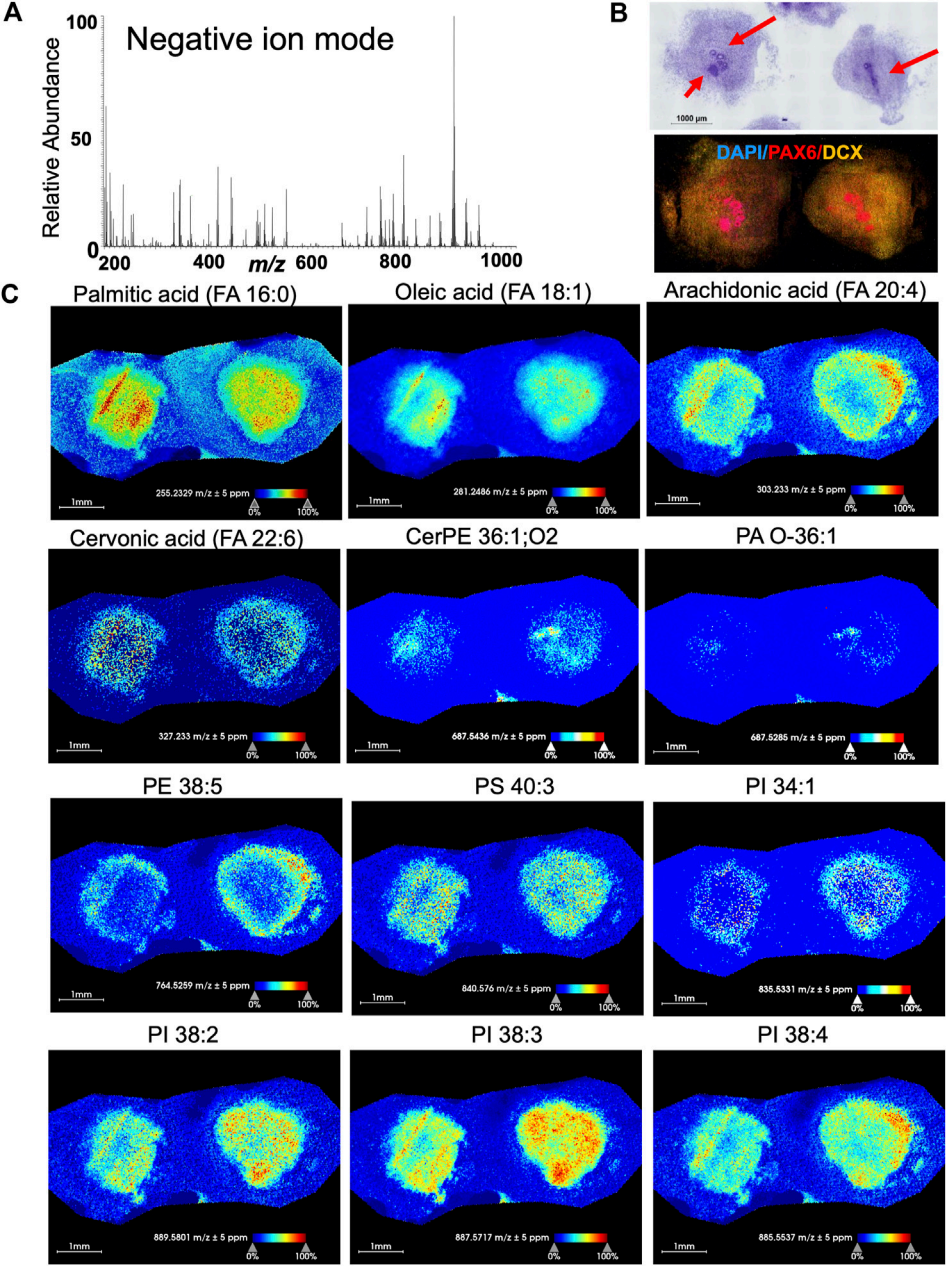
Negative ion mode MSI from 60-day-old brain organoids shows different spatial distribution and abundance of lipid species. **(A)** Average m/z spectra generated from the negative ion mode of 60-day-old brain organoids detected 168 lipids across the measured mass range of m/z 200-1,000. This includes fatty acids (m/z 200–600) and phospholipids (m/z 700–1,000) lipid signatures in negative polarity. **(B)** Hematoxylin and eosin (HE) staining along with confocal micrograph of serial sections shows organoid morphology and expression of DAPI (nuclear stain), PAX6 (marker of neuroprogenitors localized in rosettes) and doublecortin (DCX, marker of immature neurons). Arrows point to rosettes. **(C)** Ion distribution images of fatty acids and other lipids that are visualized and detected in negative ion mode represent several lipid classes including fatty acids and glycerophospholipids. All images were generated with a bin width of *Δm/z* = 0.01 at a mass resolving power of 70,000 at m/z = 200 and a mass accuracy within ±5 ppm. The spatial resolution was set to 25 µm/pixel. Each dot on the image is one pixel. The scale bar represents 1 mm and ion intensities are normalized to the total ion count (TIC) and scaled from 0% to 100%. CerPE, ceramide-phosphoethanolamine; PA, phosphatidic acid; PE, phosphatidyl-ethanolamine; PS, phosphatidyl-serine; PI, phosphatidyl-inositol.

We have detected and visualized 114 lipids of glycerophospholipid and sphingolipid classes in 60-day old organoids in positive mode and 168 species from fatty acyls, glycerophospholipid, and sphingolipid classes in negative mode within the mass accuracy of ±5 ppm (Supplementary Tables S1, S2). In positive ion mode, we have considered lipids with three different ion adducts (protonated [H^+^], sodiated [Na^+^], and potassiated [K^+^]) and in negative ion mode, deprotonated [H^−^] lipids. The lipids were measured within the mass range of m/z 200-1,000 and images were acquired with a pixel size of 25 × 25 µm. We depict representative samples in Figure 3 (positive mode) and Figure 4 (negative mode) along with the average spectrum of all lipid signatures at both polarities (Figures 3A, 4A, respectively; the m/z of each species shown in Figures 3C, 4C, respectively, is listed) and histology of adjacent sections (Figures 3B, 4B).

Different lipid classes have different distributions within the organoid tissue (Figures 3, 4). In positive mode, lysophosphatidyl-choline (LPC) species were abundant throughout the tissue but at different concentrations (Figure 3C). Higher abundance was observed at the organoid poles, where cortical neurons are present (doublecortin [DCX] staining in Figure 3B). In contrast, phosphatidyl-ethanolamine (PE) and phosphatidyl-choline (PC) species, while also different in abundance, were more uniformly distributed within the organoids, unlike phosphatidyl-serine PS 40:6 that was present only around the organoid edges.

In negative mode, we observed different fatty acids. Palmitic acid was the most abundant particularly toward the center of organoid (Figure 4C). In contrast, arachidonic acid was observed mostly on the outer edges. Phosphatidyl-inositol (PI) species were mostly distributed throughout the organoid, and those with 38 carbons were particularly abundant. Interestingly, phosphatidic acid PA O-36:1 was almost not observed (Figure 4C). These data show for the first time the abundance and the distribution of different lipids in human 60-day-old brain organoids, prompting further studies of the relevance of specific lipids, their cell-type specificity, and their role in organoid development and maturation.

### Lipid signatures of neurogenic niches in brain organoids

**FIGURE 5:**
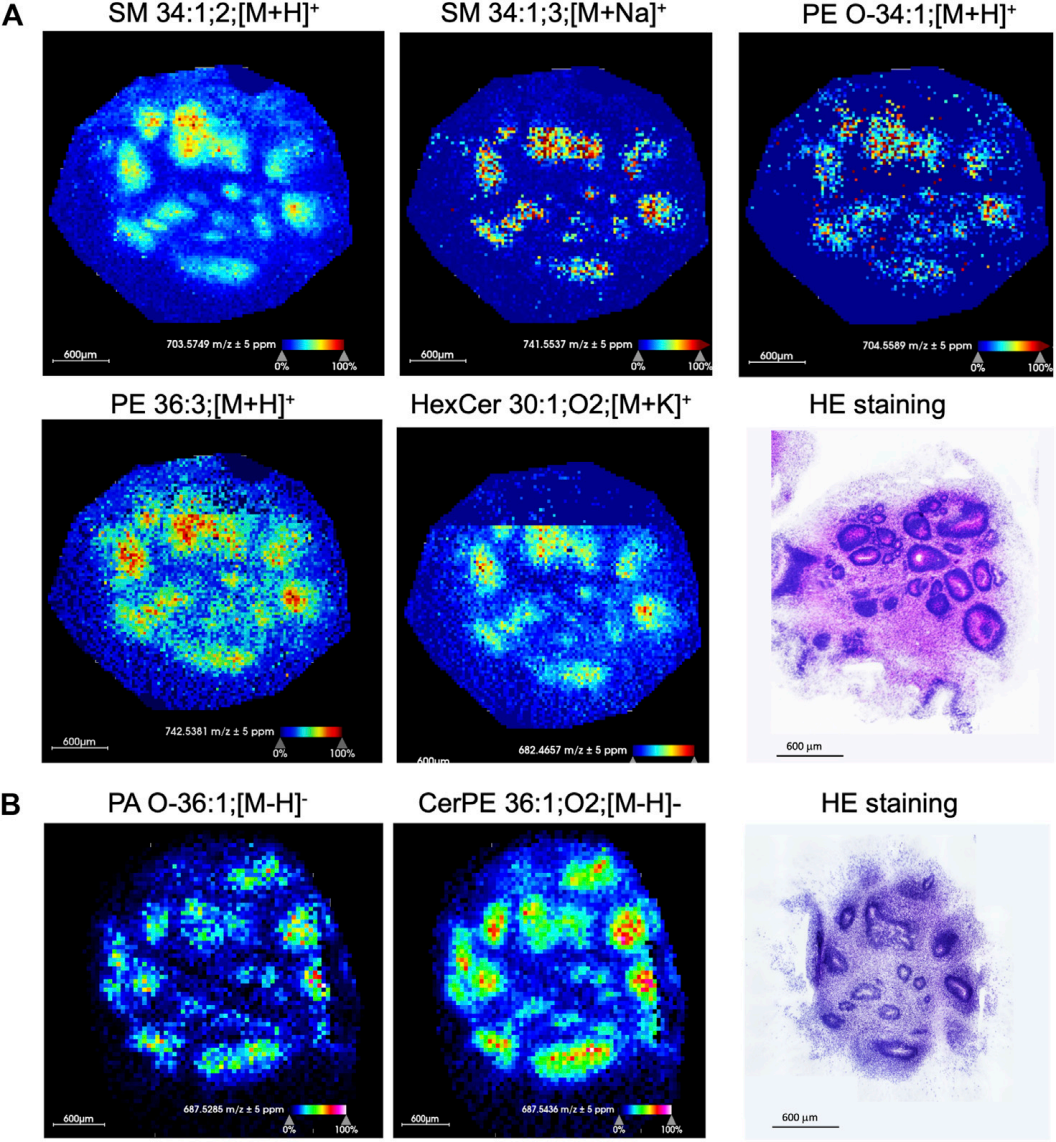
Specific lipids are enriched in rosettes. **(A)** Certain sphingolipids and phospholipids are selectively distributed in rosettes of a 60-day-old brain organoid. Ion distribution was acquired in positive mode. The HE staining of a serial organoid section shows histological architecture of rosettes. **(B)** Ion distribution of deprotonated PA O-36:1 and 2-hydroxylated CerPE 36:1 in negative mode shows selective localization in rosettes. The HE staining of a serial organoid section shows histological architecture of rosettes. All images were generated with a bin width of *Δm/z* = 0.01 at a mass resolving power of 70,000 at m/z = 200 and a mass accuracy within ±5 ppm. The spatial resolution was set to 25 µm/pixel. Each dot on the image is one pixel. The scale bar represents 600 um and ion intensities are normalized to the total ion count (TIC) and scaled from 0% to 100%. SM, sphingomyelin; PE, phosphatidyl-ethanolamine; HexCer, hexosylceramide; PA, phosphatidic acid; CerPE, ceramide-phosphoethanolamine.

**FIGURE 6:**
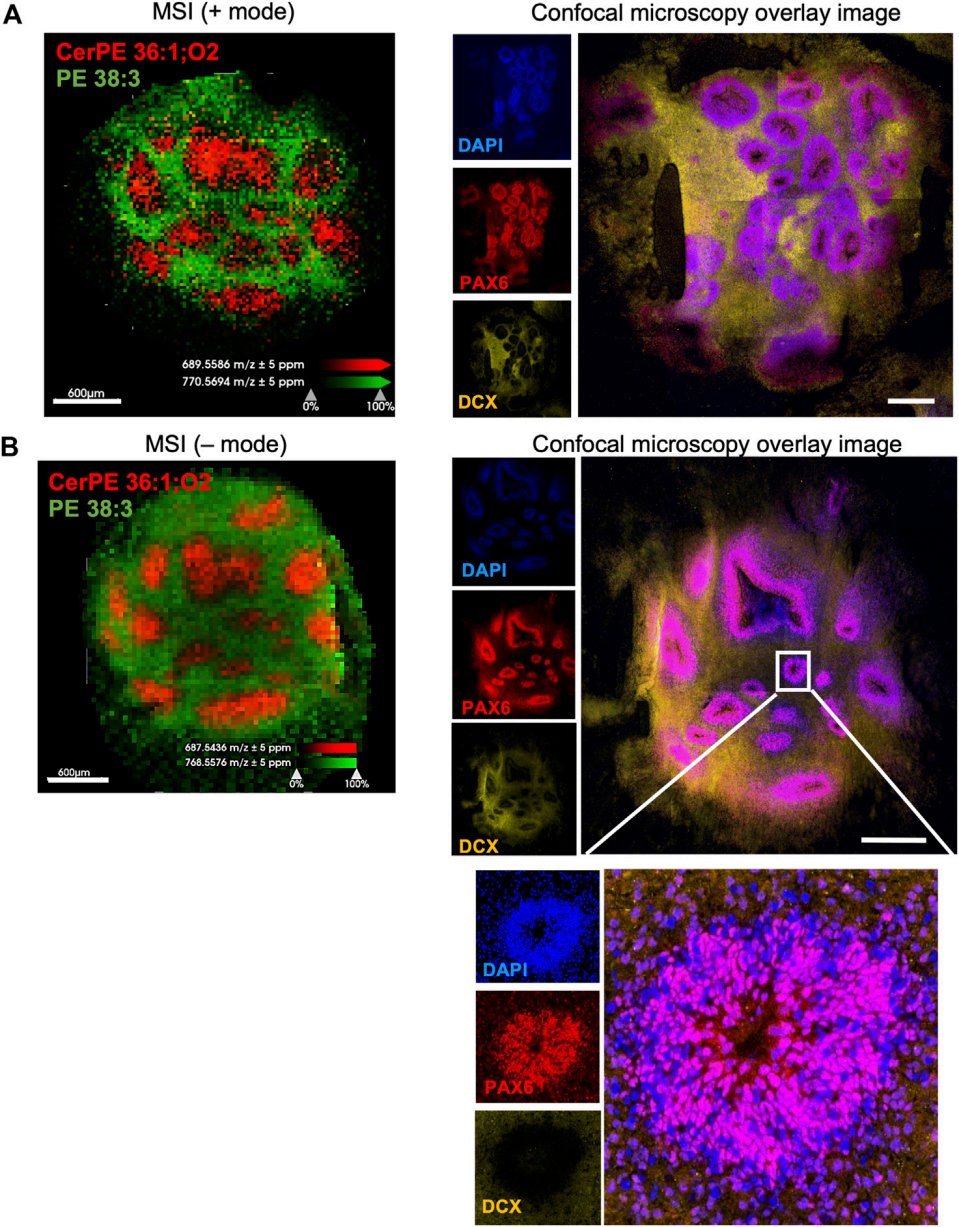
Sphingolipid CerPE 36:1; O2 is selectively abundant in rosettes, while PE 38:3 is selectively absent from rosettes. Positive **(A)** and negative **(B)** ion mode shows overlaid distribution of CerPE 36:1; O2 and PE 38:3 in two 60-day-old brain organoids. Confocal micrographs of sections adjacent to the sections used for MSI show expression of DAPI (nuclear stain), PAX6 (marker of neuroprogenitors localized in rosettes) and doublecortin (DCX, marker of immature neurons). Inset in **(B)** is a high magnification of a rosette, showing expression of PA×6 and not DCX.

At 60 days, brain organoids consist of rosettes—flower-shaped clusters of neuro-progenitors organized around a central hole—and different neuroepithelial progeny such as neurons and astrocytes, that differentiated from neuroprogenitors. Rosettes differ from other organoid regions not only by structure and cellular content but also by their function as they are the sites of neuroprogenitor proliferation, neurogenesis and astrogenesis. As such, they are of particular interest because metabolic processes that drive proliferation are different from those that drive differentiation and maturation of postmitotic cells. Interestingly, our MSI analysis has distinguished seven lipid signatures, ranging between m/z 600–800, that were selectively abundant in rosettes. These included sphingomyelins (SM 34:1;2, SM 34:1;3), phosphatidyl-ethanolamine (PE O-34:1, PE 36:3), phosphatidic acids PA O-36, and ceramides (HexCer 30:1;O2, CerPE 36:1;O2) (Figure 5). Those specific lipids are present in the nervous system and are important for brain development and function including cell growth, differentiation, and apoptosis (Cermenati et al., 2015). We observed a particularly striking distribution of ceramide-phosphoethanolamine CerPE 36:1; O2 which was restricted within rosettes and of phosphatidyl-ethanolamine PE 38:3, which was distributed throughout the organoid tissue but not in rosettes (Figures 6A, B). This suggests that ceramide in this particular lipid species might be important for neuroprogenitor biology, while its removal may be important for terminal differentiation of their progeny (see the insert in Figure 6B that highlights presence of PAX6 expressing neuroprogenitors and absence of DCX expressing immature neurons in the rosette). Differential distribution of lipids in brain organoids may thus represent different cellular functions corresponding to brain lipid metabolism during neurodevelopment and in disease (Veloso et al., 2011).

## Discussion

In this paper, we present an optimized protocol for HR-MALDI-MSI of human brain organoids and first data on the lipid distribution in these models. While the technology has not reached a single-cell level, we can still make important conclusion about lipid species localization based on the histological analyses of the tissue. This is particularly true for the neurogenic regions within the organoids, which are easily identified based on staining and represent the source of all other cell types in the organoid. Our HR-MALDI-MSI of brain organoids is capable of mapping molecular distribution of lipid classes such as glycerophospholipids, sphingolipids, and fatty acids. This can help identify key signaling pathways, metabolic processes, and other cellular mechanisms involved in brain development and function. Additionally, mass spectrometry can provide information on the location and distribution of specific molecules within the organoid, which can be useful for understanding the spatial organization of 3D models of the human brain.

In recent years, significant progress has been made in MSI technology, which has evolved as a powerful bioanalytical tool for identifying, quantifying, and visualizing the distribution of various molecules in biospecimens with high mass accuracy and high sensitivity. By using MSI to study brain organoids, we can gain insights into the cellular and molecular processes that govern early brain development and function. However, metabolic imaging of biological tissue is still in an infant stage as there is a critical need for proper sample preparation and instrument settings. A suitable preparation routine must preserve the native state of the tissue concerning morphology, chemical composition, and distribution of the analytes, remove excess salts from culture media, and be reproducible.

The protocol we propose for MSI of brain organoids is based on reagents and compounds that can decrease background interference with the imaging platform and is supported by imaging studies of other organoids (Bakker et al., 2022). Flash-freezing the samples is optimal not only because it avoids many preparations (e.g., formalin fixation) but also because it does not cause cross-linking of molecules, making them unavailable for ionization. Further, we do not recommend use of the polymer polyethylene glycol, a frequent embedding media for storage of organoids prior to sectioning, because it can interfere with the matrix and contaminate peaks in the mass spectra (Buchberger et al., 2018). Based on our data using three different embedding materials, we recommend 10% gelatin from fish skin as an embedding media (Gill et al., 2017) because it produced the best solution for cryostat sectioning and MSI of brain organoids. Organoids were thinly sectioned (14 µm thickness) and thaw-mounted onto the appropriate surface (ITO or microscope slides) for MSI and histological studies. One of the major optimizations we made was to limit delocalization of lower m/z lipids such as fatty acids assigned at negatively charged ion images, evident when comparing the m/z images generated from the optimized fish gelatin embedding protocol to images obtained from water and porcine gelatin embedding. Through careful optimization from the moment of tissue fixation to data collection and analysis, we ensured high-quality, high sensitivity, and reproducibility of brain organoid data using MSI.

The first data gathered from our studies focus on discovery of lipid distributions in the tissue. Lipids in the central nervous system not only have structural and energy-production roles but also act as intracellular signaling molecules (Kandel et al., 2022). Our data point to seven lipids (SM 34:1;2, SM 34:1;3, PE O-34:1, PE 36:3, HexCer 30:1;O2, PA O-36:1, CerPE 36:1) that could differentiate histologically-confirmed rosettes from other parts of the brain organoid, populated by neurons and astrocytes.

Among the sphingolipids enriched in rosettes, two sphingomyelin (SM) molecules were identified, SM 34:1;2 and SM 34:1;3. These denote SM species with a total of 34 carbons (a sphingosine backbone C18:0 and fatty acid of C16), 1 double bond, and 2 or 3 hydroxyl groups in the ceramide backbone, respectively. SM 34:1;2 is almost absent from neurons and oligodendrocytes (Fitzner et al., 2020) and was recently detected in the cerebrospinal fluid derived from patients affected by demyelination (Péter et al., 2020). Interestingly, SM 34:1 was detected by HR-MALDI-MSI in the mouse dentate gyrus area, the site of adult neurogenesis (Škrášková, 2016), and it was one of major SMs present in the developing rat brain (Löhmann et al., 2010). This concordant cross-species abundance of the SM 34:1 in the neurogenic zones further validates our data. Although the specific function of each molecular species of SM is unknown, in general these molecules are involved in the formation of lipid rafts, cell signaling, lipid and protein sorting, and membrane trafficking (Chakraborty and Jiang, 2013). In particular, the SM 34:1 is found especially on the cell membrane outer leaflet, where it can change the plasma membrane curvature-elastic parameters and structural organization (Arkhipov et al., 2013). This function has been attributed to the presence of the amide bonded acyl chain of 16 carbons, associating SM 34:1 with reduced membrane bilayer thickness (Niemelä et al., 2006) and the function of some membrane proteins (Niemelä et al., 2006; Mencarelli and Martinez-Martinez, 2013). Sphingomyelinase-2 (or sphingomyelin phosphodiesterase 1, SMase) is an enzyme that breaks down SMs into ceramides. Inhibition of SMase by fluoxetine leads to the build-up of SM 34:1, which has been considered to be neuroprotective (Lim et al., 2009; Kim et al., 2007), whereas hyperactivation of SMase has been associated with generation of very long ceramides that promote neural differentiation (Bi et al., 2021; Aguirre et al., 2005; He et al., 2014). Interestingly, SM 34:1 has previously been suggested to represent lipidomic signature of longevity, associated with low activity of SMase (Montoliu et al., 2014).

Phosphatidylethanolamine PE 36:3 was clustered in rosettes as well. Previously, it was reported to be enriched in the mouse hippocampus (Hu et al., 2022) and high levels of PE 36:3 were found in cultured neuroprogenitors of control and autism patients after fluoxetine treatment (Arora, 2022). Finally, another abundant sphingolipid in rosettes was CerPE 36:1. This understudied structural analog of SM, which substitutes an ethanolamine group for the choline group of SM (Carvalho et al., 2012), is a major membrane sphingolipid in invertebrates, such as *Drosophila*, and in some bacterial species. Small amounts of CerPE have also been detected in mammals (van Echten-Deckert and Herget, 2006), specifically in ganglionic eminence (Bhaduri et al., 2021; Ding et al., 2015), and it was increased in Parkinson disease (Beger et al., 2022). CerPE is considered critical for meiotic cytokinesis (Kunduri et al., 2022) and it might support the mitotic potential of rosette neuroprogenitors. We also found phospholipids such as plasmenyl phosphatidylethanolamine PE O-34:1, ceramides HexCer 30:1;O2, and plasmenyl phosphatidic acid PA O-36:1 in rosettes. These lipids may play a role in the development and maintenance of rosettes (Udagawa and Hino, 2022).

Overall, metabolome localization in brain organoids using MSI is a promising area of research that has the potential to shed new light on the complex processes that govern brain development by identifying specific lipid signatures, metabolic routes, and metabolic cell fate trajectories. As technology continues to improve and become more accessible, it is likely that we will see an increasing number of studies using MSI for spatial metabolomics of brain organoids and other complex biological systems.

## Data Availability

The datasets presented in this study can be found in online repositories. The names of the repository/repositories and accession number(s) can be found below: MBL-EBI MetaboLights database www.ebi.ac.uk/metabolights/MTBLS7565.

## Supplementary material

SUPPLEMENTARY TABLE S1 Lipid species in brain organoids detected in positive mode and in both positive and negative mode.

SUPPLEMENTARY TABLE S2 Lipid species detected from brain organoids sections in negative mode.
