# Supplementary material for: Mass spectrometry imaging as an emerging tool for studying metabolism in human brain organoids
Source: Front Mol Biosci. 2023 May 22;10:1181965. doi: 10.3389/fmolb.2023.1181965 (PMC10251497; doi:10.3389/fmolb.2023.1181965)
Supplement: Supplementary file 1 [file DataSheet2.PDF]

Assighnment	Chemical formula	Theoretical m/z	Observed m/z	Mass accuracy (ppm)	Ion
FA 16:1	C16H30O2	253,2173	253,2176	1,2	[M-H]-
FA 16:0	C16H32O2	255,2330	255,2327	-1,0	[M-H]-
FA 18:3	C18H30O2	277,2173	277,2175	0,7	[M-H]-
FA 18:2	C18H32O2	279,2330	279,2330	0,2	[M-H]-
FA 18:1	C18H34O2	281,2486	281,2483	-1,1	[M-H]-
FA 18:0	C18H36O2	283,2643	283,2646	1,2	[M-H]-
FA 20:4	C20H32O2	303,2330	303,2332	0,8	[M-H]-
FA 20:3	C20H34O2	305,2486	305,2489	1,0	[M-H]-
FA 20:0	C20H40O2	311,2956	311,2957	0,5	[M-H]-
FA 22:6	C22H32O2	327,2330	327,2330	0,2	[M-H]-
FA 22:5	C22H34O2	329,2486	329,2488	0,6	[M-H]-
FA 22:4	C22H36O2	331,2643	331,2643	0,2	[M-H]-
FA 22:3	C22H38O2	333,2799	333,2797	-0,6	[M-H]-
FA 22:2	C22H40O2	335,2956	335,2956	0,1	[M-H]-
FA 22:1	C22H42O2	337,3112	337,3114	0,6	[M-H]-
LPE 18:1	C23H46NO7P	478,2965	478,2959	-1,3	[M-H]-
LPE 18:0	C23H48NO7P	480,3100	480,3096	-0,8	[M-H]-
LPG 16:0	C22H45O9P	483,2717	483,2729	2,4	[M-H]-
LPE 20:4	C25H44NO7P	500,2772	500,2783	2,3	[M-H]-
LPG 18:1	C24H47O9P	509,2874	509,2885	2,2	[M-H]-
LPS 18:1	C24H46NO9P	522,2826	522,2838	2,2	[M-H]-
LPG 20:4	C26H45O9P	531,2717	531,2729	2,2	[M-H]-
Cer 34:1;O2	C34H67NO3	536,5010	536,5018	1,5	[M-H]-
Cer 34:0;O2	C34H69NO3	538,5193	538,5205	2,2	[M-H]-
LPG 22:6	C28H45O9P	555,2717	555,2729	2,1	[M-H]-
Cer 36:1;O2	C36H71NO3	564,5323	564,5361	6,7	[M-H]-
LPI 18:0	C27H53O12P	599,3191	599,3202	1,9	[M-H]-
PA 32:2	C35H65O8P	643,4333	643,4344	1,7	[M-H]-
PA 32:1	C35H67O8P	645,4490	645,4501	1,7	[M-H]-
PE O-30:1	C35H70NO7P	646,4778	646,4767	-1,7	[M-H]-
PE 30:1	C35H68NO8P	660,4620	660,4610	-1,5	[M-H]-
PE 30:0	C35H70NO8P	662,4779	662,4766	-2,0	[M-H]-
PE O-32:2	C37H72NO7P	672,4978	672,4974	-0,6	[M-H]-
PA 34:1	C37H71O8P	673,4811	673,4814	0,4	[M-H]-
CerPE 34:1;O3	C36H73N2O7P	675,5104	675,5083	-3,1	[M-H]-
PE 32:2	C37H70NO8P	686,4768	686,4766	-0,3	[M-H]-
PA O-36:1	C39H77O7P	687,5285	687,5274	-1,6	[M-H]-
CerPE 36:1;O2	C38H77N2O6P	687,5436	687,5447	1,7	[M-H]-
PE 32:1	C37H72NO8P	688,4912	688,4923	1,6	[M-H]-
PE 32:0	C37H74NO8P	690,5041	690,5059	2,6	[M-H]-
PE O-34:3	C39H74NO7P	698,5160	698,5130	-4,3	[M-H]-
PA 36:1	C39H75O8P	701,5116	701,5127	1,6	[M-H]-
PE O-34:1	C39H78NO7P	702,5455	702,5443	-1,7	[M-H]-
PA 36:0	C39H77O8P	703,5234	703,5283	7,0	[M-H]-
PE 34:4	C39H70NO8P	710,4734	710,4766	4,5	[M-H]-

PE 34:3	C39H72NO8P	712,4945	712,4923	-3,1	[M-H]-
PE 34:2	C39H74NO8P	714,5068	714,5079	1,5	[M-H]-
PE 34:1	C39H76NO8P	716,5225	716,5236	1,6	[M-H]-
PA O-38:0	C41H83O7P	717,5755	717,5746	-1,3	[M-H]-
PE 34:0	C39H78NO8P	718,5363	718,5392	4,0	[M-H]-
PG 32:1	C38H73O10P	719,4890	719,4869	-2,9	[M-H]-
PE O-36:6	C41H72NO7P	720,4967	720,4974	1,0	[M-H]-
PG 32:0	C38H75O10P	721,5014	721,5025	1,5	[M-H]-
PE O-36:5	C41H74NO7P	722,5085	722,5076	-1,2	[M-H]-
PE O-36:4	C41H76NO7P	724,5285	724,5287	0,3	[M-H]-
PE O-36:2	C41H80NO7P	728,5616	728,5600	-2,2	[M-H]-
CerPE 38:2;O3	C40H79N2O7P	729,5545	729,5552	1,0	[M-H]-
PE O-36:1	C41H82NO7P	730,5724	730,5756	4,4	[M-H]-
PS 32:1	C38H72NO10P	732,4789	732,4821	4,4	[M-H]-
PE O-36:0	C41H84NO7P	732,5924	732,5913	-1,5	[M-H]-
PE 36:5	C41H72NO8P	736,4898	736,4923	3,4	[M-H]-
PE 36:4	C41H74NO8P	738,5061	738,5079	2,4	[M-H]-
PE 36:3	C41H76NO8P	740,5252	740,5236	-2,2	[M-H]-
PE 36:2	C41H78NO8P	742,5373	742,5392	2,6	[M-H]-
PG 34:3	C40H73O10P	743,4856	743,4869	1,7	[M-H]-
PE 36:1	C41H80NO8P	744,5550	744,5549	-0,1	[M-H]-
PG 34:2	C40H75O10P	745,5049	745,5025	-3,2	[M-H]-
PE O-38:7	C43H74NO7P	746,5122	746,5130	1,1	[M-H]-
PS O-34:1	C40H78NO9P	746,5333	746,5342	1,2	[M-H]-
PG 34:1	C40H77O10P	747,5228	747,5216	-1,6	[M-H]-
PE O-38:6	C43H76NO7P	748,5269	748,5287	2,4	[M-H]-
PS O-34:0	C40H80NO9P	748,5520	748,5498	-2,9	[M-H]-
PG 34:0	C40H79O10P	749,5316	749,5338	2,9	[M-H]-
PE O-38:3	C43H82NO7P	754,5760	754,5756	-0,5	[M-H]-
PA 40:2	C43H80O8P	755,5596	755,5586	-1,4	[M-H]-
PE O-38:2	C43H84NO7P	756,5894	756,5913	2,5	[M-H]-
CerPE 40:2;O3	C42H83N2O7P	757,5865	757,5865	0,0	[M-H]-
PS 34:2	C40H74NO10P	758,5015	758,4978	-4,9	[M-H]-
PE O-38:1	C43H86NO7P	758,6045	758,6069	3,2	[M-H]-
PE 38:7	C43H72NO8P	760,4913	760,4923	1,3	[M-H]-
PS 34:1	C40H76NO10P	760,5123	760,5134	1,4	[M-H]-
PE 38:6	C43H74NO8P	762,5073	762,5079	0,8	[M-H]-
PS 34:0	C40H78NO10P	762,5286	762,5291	0,7	[M-H]-
PE 38:5	C43H76NO8P	764,5259	764,5236	-3,0	[M-H]-
PE 38:4	C43H78NO8P	766,5430	766,5392	-5,0	[M-H]-
PE 38:3	C43H80NO8P	768,5576	768,5549	-3,5	[M-H]-
PG 36:4	C42H75O10P	769,5014	769,5025	1,4	[M-H]-
PE 38:2	C43H82NO8P	770,5741	770,5705	-4,7	[M-H]-
PG 36:3	C42H77O10P	771,5151	771,5182	4,0	[M-H]-
DGCC 36:5	C46H79NO8	772,5711	772,5733	2,8	[M-H]-
PE 38:1	C43H84NO8P	772,5834	772,5862	3,6	[M-H]-
PG 36:2	C42H79O10P	773,5366	773,5338	-3,6	[M-H]-
PE O-40:7	C45H78NO7P	774,5452	774,5443	-1,2	[M-H]-

PS O-36:1	C42H82NO9P	774,5681	774,5655	-3,4	[M-H]-
PG 36:1	C42H81O10P	775,5484	775,5495	1,5	[M-H]-
PE O-40:6	C45H80NO7P	776,5609	776,5600	-1,2	[M-H]-
PE O-40:5	C45H82NO7P	778,5738	778,5756	2,3	[M-H]-
PS 36:4	C42H74NO10P	782,4967	782,4978	1,5	[M-H]-
PS 36:3	C42H76NO10P	784,5140	784,5134	-0,8	[M-H]-
PE 40:8	C45H74NO8P	786,5056	786,5079	2,9	[M-H]-
PS 36:2	C42H78NO10P	786,5289	786,5291	0,3	[M-H]-
PE 40:7	C45H76NO8P	788,5246	788,5236	-1,3	[M-H]-
PS 36:1	C42H80NO10P	788,5436	**788,5447**	1,4	[M-H]-
PE 40:6	C45H78NO8P	790,5398	790,5392	-0,8	[M-H]-
PE 40:5	C45H80NO8P	792,5508	792,5518	1,3	[M-H]-
PG 38:6	C44H75O10P	793,5036	793,5025	-1,4	[M-H]-
PS O-38:5	C44H78NO9P	794,5344	794,5342	-0,3	[M-H]-
PE 40:4	C45H82NO8P	794,5714	794,5705	-1,1	[M-H]-
PG 38:5	C44H77O10P	795,5205	795,5182	-2,9	[M-H]-
PS O-38:4	C44H80NO9P	796,5481	796,5498	2,1	[M-H]-
PE 40:3	C45H84NO8P	796,5854	796,5862	1,0	[M-H]-
PG 38:4	C44H79O10P	797,5378	797,5338	-5,0	[M-H]-
PE 40:2	C45H86NO8P	798,6011	798,6018	0,9	[M-H]-
PS O-38:2	C44H84NO9P	800,5797	800,5811	1,7	[M-H]-
PE 40:1	C45H88NO8P	800,6127	800,6117	-1,2	[M-H]-
PG 38:2	C44H83O10P	801,5673	801,5651	-2,7	[M-H]-
PS 37:1	C43H82NO10P	802,5585	802,5604	2,4	[M-H]-
PE O-42:7	C47H82NO7P	802,5767	802,5756	-1,4	[M-H]-
PA 44:6	C47H81O8P	803,5600	803,5596	-0,5	[M-H]-
PE O-42:6	C47H84NO7P	804,5905	804,5913	1,0	[M-H]-
PG O-40:7	C46H79O9P	805,5374	805,5389	1,9	[M-H]-
PA 44:5	C47H83O8P	805,5759	805,5753	-0,7	[M-H]-
PE O-42:5	C47H86NO7P	806,6101	806,6069	-4,0	[M-H]-
PI 32:1	C41H77O13P	807,5018	807,5029	1,4	[M-H]-
PS 38:5	C44H76NO10P	808,5109	808,5134	3,1	[M-H]-
PI 32:0	C41H79O13P	809,5175	809,5186	1,4	[M-H]-
PS 38:4	C44H78NO10P	810,5280	810,5291	1,4	[M-H]-
PE 42:9	C47H76NO8P	812,5273	812,5256	-2,1	[M-H]-
PS 38:3	C44H80NO10P	812,5464	812,5447	-2,1	[M-H]-
PE 42:8	C47H78NO8P	814,5417	814,5392	-3,1	[M-H]-
PS 38:2	C44H82NO10P	814,5612	814,5604	-1,0	[M-H]-
PE 42:7	C47H80NO8P	816,5574	816,5549	-3,1	[M-H]-
PS 38:1	C44H84NO10P	816,5754	816,5760	0,7	[M-H]-
PE 42:6	C47H82NO8P	818,5726	818,5705	-2,6	[M-H]-
PG 40:7	C46H77O10P	819,5192	819,5182	-1,2	[M-H]-
PE 42:5	C47H84NO8P	820,5880	820,5862	-2,2	[M-H]-
PG 40:6	C46H79O10P	821,5367	821,5338	-3,5	[M-H]-
PE 42:4	C47H86NO8P	822,6009	822,6018	1,1	[M-H]-
PE 42:3	C47H88NO8P	824,6128	824,6175	5,7	[M-H]-
PI 34:3	C43H77O13P	831,5018	831,5029	1,3	[M-H]-
PS 40:7	C46H76NO10P	832,5174	832,5134	-4,8	[M-H]-

PI 34:2	C43H79O13P	833,5175	833,5186	1,4	[M-H]-
PS 40:6	C46H78NO10P	834,5302	834,5291	-1,3	[M-H]-
PI 34:1	C43H81O13P	835,5331	835,5342	1,3	[M-H]-
PS 40:5	C46H80NO10P	836,5481	836,5447	-4,1	[M-H]-
PI 34:0	C43H83O13P	837,5488	837,5499	1,4	[M-H]-
PS 40:4	C46H82NO10P	838,5587	838,5604	2,0	[M-H]-
PS 40:3	C46H84NO10P	840,5778	840,5760	-2,1	[M-H]-
PS 40:2	C46H86NO10P	842,5890	842,5917	3,2	[M-H]-
PS 40:0	C46H90NO10P	846,6238	846,6230	-0,9	[M-H]-
PI 36:4	C45H79O13P	857,5175	857,5186	1,3	[M-H]-
PS 42:8	C48H78NO10P	858,5335	858,5294	-4,8	[M-H]-
PI 36:3	C45H81O13P	859,5331	859,5342	1,3	[M-H]-
PI 36:2	C45H82O13P	861,5497	861,5499	0,2	[M-H]-
PS 42:6	C48H82NO10P	862,5618	862,5604	-1,6	[M-H]-
PI 36:1	C45H85O13P	863,5644	863,5655	1,3	[M-H]-
PS 42:5	C48H84NO10P	864,5786	864,5760	-3,0	[M-H]-
PI 36:0	C45H87O13P	865,5801	865,5812	1,3	[M-H]-
PS 42:2	C48H90NO10P	870,6219	870,6230	1,3	[M-H]-
PI 38:6	C47H79O13P	881,5175	881,5186	1,3	[M-H]-
PI 38:5	C47H81O13P	883,5331	883,5342	1,2	[M-H]-
PI 38:4	C47H83O13P	885,5537	885,5499	-4,3	[M-H]-
PI 38:3	C47H84O13P	887,5717	887,5716	-0,1	[M-H]-
PS 44:7	C50H84NO10P	888,5766	888,5760	-0,7	[M-H]-
PI 38:2	C47H87O13P	889,5801	889,5812	1,3	[M-H]-
PI 38:1	C47H89O13P	891,5957	891,5968	1,2	[M-H]-
PS 44:4	C50H90NO10P	894,6279	894,6250	-3,2	[M-H]-
PI 40:5	C49H85O13P	911,5697	911,5675	-2,4	[M-H]-
