# Supplementary material for: Mass spectrometry imaging as an emerging tool for studying metabolism in human brain organoids
Source: Front Mol Biosci. 2023 May 22;10:1181965. doi: 10.3389/fmolb.2023.1181965 (PMC10251497; doi:10.3389/fmolb.2023.1181965)
Supplement: Supplementary file 2 [file DataSheet1.PDF]

M_Z positive mode					
**Assighnment**	**Chemical formula**	**Theoretical m/z**	**Observed m/z**	**Mass accuracy (p**	**Ion**
LPE 16:1	C21H42NO7P	452,2775	452,2772	-0,66	[M+H]+
LPE 18:4	C23H40NO7P	474,2615	474,2596	-4,01	[M+H]+
LPE 18:3	C23H42NO7P	476,2772	476,2749	-4,83	[M+H]+
LPE 18:2	C23H44NO7P	478,2928	478,2929	0,21	[M+H]+
LPC 16:1	C24H48NO7P	494,3244	494,3241	-0,61	[M+H]+
LPC 16:0	C24H50NO7P	496,3398	496,3411	2,62	[M+H]+
LPE 20:5	C25H42NO7P	500,2772	500,2748	-4,80	[M+H]+
LPE 20:4	C25H44NO7P	502,2928	502,2904	-4,78	[M+H]+
LPC 18:4	C26H46NO7P	516,3085	516,3061	-4,65	[M+H]+
LPC 18:3	C26H48NO7P	518,3241	518,3217	-4,63	[M+H]+
LPC 18:2	C26H50NO7P	520,3398	520,3385	-2,50	[M+H]+
LPC 18:1	C26H52NO7P	522,3554	522,3531	-4,40	[M+H]+
LPC 18:0	C26H54NO7P	524,3711	524,3718	1,33	[M+H]+
LPE 22:4	C27H48NO7P	530,3241	530,3217	-4,53	[M+H]+
LPC 20:5	C28H48NO7P	542,3241	542,3217	-4,43	[M+H]+
LPC 20:4	C28H50NO7P	544,3398	544,3374	-4,41	[M+H]+
LPC 20:3	C28H52NO7P	546,3554	546,3530	-4,39	[M+H]+
PE 32:3	C37H68NO8P	686,4755	686,4731	-3,50	[M+H]+
PE O-34:1	C39H78NO7P	704,5589	704,5566	-3,26	[M+H]+
PE 34:5	C39H68NO8P	710,4755	710,4731	-3,38	[M+H]+
PC 32:5	C40H70NO8P	724,4912	724,4888	-3,31	[M+H]+
PE O-34:2	C39H76NO7PNa	724,5252	724,5257	0,69	[M+Na]+
PE O-36:5	C41H74NO7P	724,5276	724,5257	-2,62	[M+H]+
PC 30:1	C38H74NO8PNa	726,5044	726,5059	2,06	[M+Na]+
PC 32:4	C40H72NO8P	726,5068	726,5059	-1,24	[M+H]+
PA 38:3	C41H75O8P	727,5272	727,5269	-0,41	[M+H]+
PC 32:2	C40H76NO8P	730,5381	730,5391	1,37	[M+H]+
PE O-36:2	C41H80NO7P	730,5745	730,5741	-0,55	[M+H]+
SM 36:1;2	C41H84N2O6P	731,6061	731,6052	-1,23	[M+H]+
PC 32:0	C40H80NO8P	734,5694	734,5691	-0,41	[M+H]+
PE 36:6	C41H70NO8P	736,4912	736,4888	-3,26	[M+H]+
PC O-32:1	C40H80NO7PNa	740,5565	740,5537	-3,78	[M+Na]+
PC O-34:4	C42H78NO7P	740,5589	740,5622	4,46	[M+H]+
PE 34:0	C39H78NO8PNa	742,5357	742,5386	3,91	[M+Na]+
PE 36:3	C41H76NO8P	742,5381	742,5386	0,67	[M+H]+
PG 34:3	C40H73O10P	745,5014	745,4990	-3,22	[M+H]+
PC 32:3	C40H74NO8PNa	750,5044	750,5053	1,20	[M+Na]+
PC 34:6	C42H72NO8P	750,5068	750,5053	-2,00	[M+H]+
PE O-38:6	C43H76NO7P	750,5432	750,5408	-3,20	[M+H]+
PC 34:5	C42H74NO8P	752,5225	752,5253	3,72	[M+H]+
HexCer 34:1;O4	C40H77NO10Na	754,5440	754,5401	-5,17	[M+Na]+
PC 32:1	C40H78NO8PNa	754,5357	754,5401	5,83	[M+Na]+
PC 34:4	C42H76NO8P	754,5381	754,5401	2,65	[M+H]+
PC 34:3	C42H78NO8P	756,5538	756,5514	-3,17	[M+H]+
PE O-36:0	C41H84NO7PNa	756,5878	756,5910	4,23	[M+Na]+
PE O-38:3	C43H82NO7P	756,5902	756,5910	1,06	[M+H]+

PE 38:9	C43H68NO8P	758,4755	758,4731	-3,16	[M+H]+
SM 38:1;2	C43H88N2O6P	759,6374	759,6352	-2,90	[M+H]+
SM 38:1;O2	C43H87N2O6P	759,6374	759,6375	0,13	[M+H]+
PC 34:1	C42H83NO8P	760,5851	760,5856	0,66	[M+H]+
SM 36:3;O2	C41H79N2O6PK	765,5307	765,5303	-0,52	[M+K]+
PE 38:5	C43H76NO8P	766,5381	766,5354	-3,52	[M+H]+
PC O-34:2	C42H82NO7PNa	766,5721	766,5696	-3,26	[M+Na]+
PE 36:1	C41H80NO8PNa	768,5514	768,5523	1,17	[M+Na]+
PE 38:4	C43H78NO8P	768,5538	768,5523	-1,95	[M+H]+
PE O-38:7	C43H74NO7PNa	770,5095	770,5097	0,26	[M+Na]+
PE O-38:5	C43H78NO7PNa	774,5408	774,5440	4,13	[M+Na]+
PE O-40:8	C45H76NO7P	774,5432	774,5440	1,03	[M+H]+
CerPE 40:2;4	C42H84N2O8P	775,5959	775,5929	-3,87	[M+H]+
PC 34:2	C42H80NO8P	775,5960	775,5929	-4,00	[M+NH4]+
PC 36:7	C44H74NO8P	776,5225	776,5201	-3,09	[M+H]+
PE O-40:7	C45H78NO7P	776,5589	776,5606	2,19	[M+H]+
PC 36:6	C44H76NO8P	778,5381	778,5357	-3,08	[M+H]+
HexCer 36:2;O3	C42H79NO9K	780,5386	780,5375	-1,41	[M+K]+
PC 36:5	C44H78NO8P	780,5538	780,5514	-3,07	[M+H]+
PE O-40:5	C45H82NO7P	780,5902	780,5915	1,67	[M+H]+
PC 34:1	C42H82NO8PNa	782,5670	782,5687	2,17	[M+Na]+
PC 36:3	C44H82NO8P	784,5851	784,5856	0,64	[M+H]+
SM 40:2;O2	C45H89N2O6P	785,6531	785,6538	0,89	[M+H]+
SM 40:2;2	C45H90N2O6P	785,6531	785,6557	3,31	[M+H]+
PE 40:9	C45H72NO8P	786,5068	786,5044	-3,05	[M+H]+
SM 40:1;2	C45H92N2O6P	787,6687	787,6664	-2,92	[M+H]+
SM 40:1;O2	C45H91N2O6P	787,6687	787,6688	0,13	[M+H]+
PE 40:8	C45H74NO8P	788,5225	788,5201	-3,04	[M+H]+
PC 36:1	C44H86NO8P	788,6164	788,6159	-0,63	[M+H]+
PC O-38:5	C46H84NO7P	794,6058	794,6034	-3,02	[M+H]+
SHexCer 34:1;O3	C40H77NO12S	796,5239	796,5253	1,76	[M+H]+
PC 36:4	C42H82NO8PK	798,5404	798,5415	1,38	[M+K]+
PC 38:8	C46H76NO8P	802,5381	802,5357	-2,99	[M+H]+
PC 36:4	C44H80NO8PNa	804,5514	804,5536	2,73	[M+Na]+
PC 38:7	C46H78NO8P	804,5538	804,5536	-0,25	[M+H]+
PC 38:6	C46H80NO8P	806,5694	806,5670	-2,98	[M+H]+
HexCer 38:2;O3	C44H83NO9K	808,5699	808,5677	-2,72	[M+K]+
PC 38:5	C46H82NO8P	808,5851	808,5848	-0,37	[M+H]+
PC 36:6;O2	C44H76NO10P	810,5280	810,5256	-2,96	[M+H]+
PC 38:4	C46H84NO8P	810,6007	810,5983	-2,96	[M+H]+
PE 42:10	C47H74NO8P	812,5225	812,5201	-2,95	[M+H]+
PC 36:5;O2	C44H78NO10P	812,5436	812,5412	-2,95	[M+H]+
PC 38:2	C46H88NO8P	814,6320	814,6329	1,10	[M+H]+
SM 42:1;O2	C47H95N2O6P	815,7004	815,7001	-0,37	[M+H]+
PC 38:1	C46H90NO8P	816,6477	816,6467	-1,22	[M+H]+
SHexCer 36:1;O3	C42H81NO12S	824,5559	824,5564	0,61	[M+H]+
PE O-42:7	C47H82NO7PNa	826,5721	826,5723	0,24	[M+Na]+
PE 42:2	C47H90NO8P	828,6477	828,6464	-1,57	[M+H]+

PC 40:7	C48H82NO8P	832,5851	832,5827	-2,88	[M+H]+
HexCer 40:2;O3	C46H87NO9K	836,6012	836,6040	3,35	[M+K]+
PC 40:5	C48H86NO8P	836,6164	836,6140	-2,87	[M+H]+
PC 40:4	C48H88NO8P	838,6320	838,6296	-2,86	[M+H]+
SHexCer 38:1;O3	C44H85NO12S	852,5865	852,5879	1,64	[M+H]+
Hex2Cer 34:1;O2	C46H87NO13	862,6250	862,6258	0,93	[M+H]+
PC 40:3	C48H90NO8PNa	862,6296	862,6258	-4,41	[M+Na]+
PC 44:7	C52H90NO8PK	926,6036	926,6025	-1,19	[M+K]+
PC 30:1	C38H75NO8P	704,5226	704,5230	0,61	[M+H]+
Hex2Cer 32:0;3	C44H85NO14Na	874,5906	874,5862	-5,00	[M+Na]+
PC O-36:2	C44H87NO7P	772,6220	772,6215	-0,65	[M+H]+
SM 34:1;2	C39H79N2O6PNa	725,5573	725,5568	-0,69	[M+Na]+
PA O-36:1	C39H78O7P	689,5479	689,5481	0,29	[M+H]+
CerPE 36:1;O2	C38H78N2O6P	689,5592	689,5586	-0,87	[M+H]+
PS 40:6	C46H78NO10PK	874,4935	874,4924	-1,26	[M+K]+
PE 38:3	C43H80NO8P	770,5694	770,5692	-0,26	[M+H]+
SM 34:1;2	C39H80N2O6P	703,5749	703,5747	-0,18	[M+H]+
SM 34:1;3	C39H79N2O7PNa	741,5517	741,5537	2,68	[M+Na]+
HexCer 30:1;O2	C36H69NO8K	682,4652	682,4657	0,73	[M+K]+
PE O-36:2	C41H80NO7PNa	752,5565	752,5564	-0,13	[M+Na]+
Lipids detected in both ion mode					
LPE 20:4					
PE O-34:1					
PE O-36:5					
PE O-36:2					
PE 34:0					
PE 36:3					
PG 34:3					
PE O-38:6					
PE O-36:0					
PE O-38:3					
PE 38:5					
PE 36:1					
PE 38:4					
PE O-38:7					
PE O-40:7					
PE O-40:5					
PE 40:8					
PE O-42:7					
PA O-36:1					
CerPE 36:1;O2					
PS 40:6					
PE 38:3					
